# Prioritizing Technology in Pharmacy Education: A Document Analysis of Strategic Plans

**DOI:** 10.3390/pharmacy8040237

**Published:** 2020-12-11

**Authors:** Jacob D. Dresser, Paul Burmeister, Vibhuti Arya, Kyle John Wilby

**Affiliations:** 1 School of Pharmacy, University of Otago, Dunedin 9016, New Zealand; dreja072@student.otago.ac.nz (J.D.D.); bupau492@student.otago.ac.nz (P.B.); 2 College of Pharmacy and Health Sciences, St. John’s University, Queens, NY 11439, USA; aryav@stjohns.edu

**Keywords:** pharmacy, technology, system readiness, online

## Abstract

The COVID-19 pandemic has required many pharmacy programs to increase their utilization of technology or shift the course of delivery entirely online. Delivery in this setting has exposed areas in the use of technology where pharmacy programs need to improve (such as staff and student training). This study performed a document analysis of strategic plans to identify technology-related strategies and where gaps in planning currently exist. Accredited pharmacy programs in Canada and the USA were included for analysis. A total of 77 strategic plans were identified. Strategic plans were searched for the phrases: "tech", "online", "distance" and "e-learning" to identify technology-related statements. Statements relating to technology in education were coded for (1) the prioritized “action” and (2) the objective or “goal” of this strategy. Quantitative analysis of these codes revealed that the “action” was most frequently to introduce or improve technology (54.4%), and the “goal” most frequently related to enhancing teaching/course delivery/learning (34.2%). Strategic plans appeared to frequently focus on the technology itself, with little consideration for the human aspect of operating technology or readiness of programs to embrace technology. Moving forward, strategic priorities with respect to technology should be refocused towards system readiness and account for resources necessary for target user upskilling and acceptance.

## 1. Introduction

The advent of the COVID-19 pandemic has resulted in a rapid necessity for pharmacy education programs to embrace technology and, at times, to shift the entire delivery of a program into a remote online learning environment within just a few hours or days [1]. During this transition, programs have needed to consider many aspects of technology, including (but not limited to) infrastructure, user (e.g., student or staff) training/competency, policies and procedures for appropriate use/professionalism, equity, and evaluation [1,2,3]. Online assessments delivered remotely have also needed to be managed in terms of policies and procedures, academic integrity, student competency with the assessment platform, and modification of assessment tools as needed [4,5]. Many different aspects of technology have been utilized, including live session delivery platforms, use of recordings or online information, online education delivery platforms, simulation through remote online interactions, and virtual experiential training and patient care. Despite many success stories of how quickly and effectively programs managed this transition, the experience of using technology for program delivery as a result of the COVID-19 pandemic has undoubtedly exposed gaps in programs and institutional planning processes for the effective incorporation of technology into program components [6].

One gap commonly cited in the literature and also experienced during the COVID-19 pandemic is the readiness of students and staff for technology in program delivery [7,8]. Barriers related to access, infrastructure, technology competency, and resistance to change have all been identified as limitations to the readiness of programs to adopt technology within the teaching, learning, and assessment space [8,9]. Outside of the forced use of technology experienced during the COVID-19 pandemic, successful implementation of any technology-enhanced learning event or assessment is dependent on whether or not there is adequate resourcing, organizational support, local expertise, infrastructure, and student/staff buy-in [10,11]. Moving forward through the COVID-19 pandemic and afterward, programs will need to develop clear strategies for the incorporation, use, and evaluation of technology for program delivery and assessment [12,13]. Programs will then need to outline specific goals and objectives of how they can ensure their systems are ready for technology-enhancement and the changes that come with it.

Strategic planning around the incorporation of technology into health professional programs is not well studied. The literature to date is focused on institutional strategies, which may not be relevant to health professional programs, including pharmacy [14,15,16]. What the literature does suggest, however, is that strategic planning for technology should be flexible and must account for rapid change. For example, a strategic plan meant to be relevant for five years could be quickly outdated as technology progresses and changes the way programs operate. Strategic plans should also move beyond the input of technology into a program and should address the issues described above, such as the human factors required to make technology successful [11,17]. Allocating resources towards effective use of technology by users could be an important strategic priority to support the achievement of intended goals. The ways in which strategic plans for pharmacy programs account for human factors with respect to the use of technology is currently unknown.

The recent increase in the use of technology during the COVID-19 pandemic is going to lead to a “new normal” and will change the way programs deliver learning material, assess students, and operate. As such, strategic planning will be required to direct future use of technology within programs, including aspects related to input, processes, output, and evaluation. The purpose of this study was, therefore, to characterize current strategic planning around technology within pharmacy programs and to identify gaps in strategic planning for technology as a whole. We aimed to answer the following research questions:

1. How do pharmacy programs prioritize education-related technology within strategic plans?
2. What goals do pharmacy programs aim to achieve with education-related technology?

## 2. Materials and Methods

### 2.1. Study Design

This was a document analysis of strategic planning data extracted from publicly available program websites [18].

### 2.2. Data Collection

A scoping search of pharmacy program websites across global geographical regions was conducted to determine the availability of strategic plans. It was determined from this search that programs in the United States and Canada were the only ones consistently publishing strategic plans on publicly available websites. This is likely due to accreditation standards for the United States and Canada requiring publication of strategic plans [19,20]. The study was therefore limited to programs in these jurisdictions. Those universities with an entry-to-practice degree in pharmacy fully accredited by the Canadian Council for Accreditation of Pharmacy Programs (CCAPP) or the Accreditation Council for Pharmacy Education (ACPE) were considered for analysis. Accredited schools were identified via the CCAPP or ACPE websites [21,22]. One investigator (K.J.W.) reviewed each linked program’s website and searched for a program’s publicly available strategic plan. The search was conducted manually by clicking on links such as “About Us”, “Accreditation”, “Mission/Vision/Goal”, or “Strategic Plan”. Keyword searches of websites were also conducted if no strategic plan could be located through manual searching. A strategic plan was included for analysis if it was specific to the pharmacy program/school/college (e.g., not a general university strategic plan) and if the information contained goals or objectives aside from a mission or vision statement.

### 2.3. Data Extraction

All included strategic plans were screened independently by two investigators (J.D.D. and P.B.). Each document was screened using the find function in Microsoft^®^ Word^®^ or PDF viewer in Google Chrome for the phrases: “tech”, “online”, “distance”, or “e-learning”. Any statement relating to the use of technology to facilitate education or program operations were extracted into a Microsoft^®^ Excel^®^ spreadsheet. Any statement specific for technology solely within the realm of research was excluded. A third investigator (K.J.W.) manually screened 10 strategic plans to ensure the find function accurately identified all statements of interest. Furthermore, one investigator (J.D.D.) completed a post hoc validation exercise by searching the terms "m-learning", "cyber", "smart", "blended", and "digital". No further statements were identified via either of these validation exercises. Three investigators (J.D.D., P.B., K.J.W.) met in person to review the independently extracted data and resolve discrepancies through discussion. At this point, any statement that was not framed as a goal or objective was marked for exclusion (e.g., statements of technology use in the introduction/preamble sections of documents).

### 2.4. Data Analysis

Two investigators (J.D.D., K.J.W.) met to review approximately 25% of the dataset to develop an initial coding framework specific to the research questions. J.D.D. and P.B. then independently coded all data according to this framework. The investigator team then met once again to review coding. At this time, the framework was further modified based on the first round of coding. Two investigators (J.D.D., P.B.) then re-coded all data according to the final stable framework. Each statement was coded twice according to each research question. Discrepancies were once again resolved during an in-person meeting using discussion (J.D.D., P.B., K.J.W.).

Once all coding was complete, investigators met to combine related codes into categories according to similarity. Investigators then completed a quantitative analysis and a qualitative analysis. For the quantitative analysis, descriptive statistics were used to summarize the proportion of strategic plans identified that prioritized technology, the median and range of statements relating to technology for each strategic plan, and the proportion of statements coded per category for the final coding framework. For the qualitative analysis, investigators met to interpret themes from the coded data. All investigators agreed upon final themes for inclusion as a result. All statistics were completed using Microsoft^®^ Excel^®^.

## 3. Results

A total of 142 program websites were searched for strategic plans. From these websites, a total of 77 (54.2%) had a publicly available strategic plan. Of those identified, 41 (53.2%) had at least one goal or objective specific to technology for education or program operations. The median number of goals/objectives relating to technology per strategic plan was 2 (range 1–5). Extracted statements from strategic plans are provided in Appendix A.

### 3.1. Strategic Priorities for Technology in Pharmacy Education

The proportion of statements coded for each prioritized action relating to technology is provided in Table 1. The majority of programs (54.4%) aimed to introduce or improve the use of technology within the program, while the remaining aimed to acquire (16.5%), prepare (15.2%), or evaluate (13.9%) technology. Statements coded for “acquire” were focused on obtaining resources or infrastructure for the successful implementation of technology. Those that prioritized introducing or using technology appeared to view technology as a new concept that could be used to enhance the educational program. Programs that strategized improving or expanding technology aimed to enhance or build on current or existing uses of technology. Preparedness strategies focused on training of staff and/or students and ensuring the end-users were ready to use technology effectively. Strategies that focused on evaluation aimed to determine the effectiveness and impact of technology within the educational program.

### 3.2. Strategic Goals for Technology in Pharmacy Education

The final coding framework for strategic goals consisted of five categories: education, practice, staff training, communication, and others. Coding results are provided in Table 2. The majority of statements were coded under education (71%), followed by practice (10%), communication (9%), other (6%), and staff development (4%). For education, 27 statements were coded as teach/deliver/learning. These statements related to improving teaching methods, how a course was delivered, or enhancing students’ learning through the use of technology. Twelve statements were coded as general, which typically referred to somehow improving an educational program, but without specifying through what means (e.g., teaching, learning, course development, etc.) The nine statements coded as online/distance courses or degrees specifically referred to the development or enhancement of distance or online-based courses or programs. The remaining eight statements categorized as “other” were coded as collaboration/IPE, student engagement/active learning, or assessment.

Goals relating to practice were distributed across four codes of general, patient care, life-long learning, and collaboration. Communication codes focused on improving operations through faculty communication, student communication, as well as using technology to create links with alumni. The other category consisted of goals coded to improve or maintain technology within a program. Goals relating to staff development were the least coded and focused on the need for training and technology acceptance.

### 3.3. Thematic Analysis

Investigators interpreted three primary themes from the data analyzed:

1. Technology is being strategically prioritized to enhance the learning experience for students

This theme is represented by the majority of statements being coded as “education” or within other categories, but for enhancing the learner experience (e.g., communication). Strategic priorities rarely branched outside of institution-controlled programs and rarely mentioned outcomes relating to broader goals, such as patient care. With respect to the learner experience, technology was strategically prioritized to improve existing programming or build new degrees/programs (e.g., online or distance degrees).

2. Strategic prioritization of technology is technology-focused and not person-focused

This theme is represented by the strong focus of strategic planning on the incorporation of technology, rather than workforce development for technology literacy or competent use. Statements primarily mentioned components of program delivery (e.g., teaching, assessment) but failed to acknowledge the users and human factors expected with increased technology use. In other words, acquiring and implementing technology was explicitly stated as the overarching strategic priority, without recognition of human factors required for effective use.

## 4. Discussion

The purpose of this study was to characterize current strategic planning around technology within pharmacy programs and to identify gaps in strategic planning for technology as a whole. Findings show that programs are mostly prioritizing the introduction of technology, as well as improvement or expansion. Fewer programs are currently prioritizing acquiring technology, preparing staff and/or students for use, or evaluation of effectiveness or impact. Findings also show that strategic goals are primarily focused on improving teaching, delivery, and learning or are general to education as a whole. Fewer programs are developing strategic goals to prepare staff or students for the use of technology or using technology as part of programs to improve practice or communication. These findings can be used to inform future strategic planning with respect to technology, especially given the recent events of the COVID-19 pandemic.

This study found that strategic planning by pharmacy programs prior to the COVID-19 pandemic was focused almost solely on the implementation or expansion of technology within programs rather than preparing users for use or evaluating effectiveness. The greatest concern about this approach is that the act of introducing or expanding existing technology within a program will be meaningless if it is ineffective, unusable or unable to be easily adopted by the faculty or students, either due to lack of training or intentional time focused on becoming familiar with the technology [16,17]. Adding technology into a program can be expensive and require other resources such as time and increased staffing. It was, therefore, surprising that the majority of strategic priorities identified aimed to simply “add” or “add more”. Moving forward, programs should strategically prioritize evaluation of the use of technology and/or include evaluation as a priority alongside any other priorities relating to the introduction of improvements. Programs should also be encouraged to build on what they have and innovate using existing technologies and skillsets, rather than requiring frequent training for the introduction of new technology unfamiliar with users.

The lack of strategic priorities and goals focused on preparing users and/or training outcomes clearly shows that pharmacy programs are not yet thinking about prioritizing what we propose to be a “systems readiness” approach when faced with forced change or even when changes may occur due to use of new platforms or innovations. In the recent pandemic, both educators and students juggled priorities while adapting to sharing workspaces with others within a household with potential space restrictions, balancing childcare, homeschooling, and/or elder care, and shifting lecture materials to remote learning using new or existing technology that may not have been regularly employed pre-COVID. If programs can integrate the use of technology into normal operations (e.g., hosting quarterly or more frequent staff meetings remotely, using technology or using lecture recordings as part of normal class lessons), staff and students alike may be more familiar and savvy with the technology in case they need to quickly shift to remote learning in the future. Dealing with individual/familial barriers to remote teaching/learning is even more complicated if technology is another barrier that requires adaptation. Additionally, though the use of technology may be promising in education, several factors must be considered so that students and staff alike are able to accommodate the “shifting” of in-person learning activities to completely remote. Some of these factors include living in a multi-generational household, living in shared spaces, potential vulnerability associated with exposure to one’s personal/household situations. As discussed above, the effectiveness of technology is dependent on usability, which directly relates to staff and student training. The findings of this study align with recent research that calls for training of staff and students in order to enhance effectiveness and ensure intended goals are met [7]. Findings also support calls from others that suggest strategic planning for staff training and development may help to facilitate change [8]. In light of these findings, pharmacy programs should reassess their strategic planning priorities and begin to recognize and acknowledge the human side of implementing or enhancing technology for education.

### 4.1. Implications

The findings of this study have implications for strategic planning, as well as future research. Coming out of the COVID-19 pandemic, programs must begin strategically planning for “system readiness” and ensuring that their users (staff/students) are able to competently use any technology that is implemented or improved/expanded within a program. Key performance indicators should be developed to outline the target number of sessions per year for staff and student engagement in technology (e.g., numbers of meetings and teaching events to be delivered online per year) to ensure users are ready-to-use online platforms in the case of a pandemic or other disruptive event. A second recommendation for strategic planning is to intentionally consider, not discount, the human factors in strategic prioritization. Although we may be able to assume good intentions for consideration of this, explicitly stating such consideration must be present to emphasize and ensure sustainability for planning efforts, and lack of such explicit statement can mean that this is easily overlooked during foundation planning discussions. Almost all strategic statements identified were focused on the technology itself and not the user or human factor. It can be easily argued that introducing or expanding technology without proper user training will undercut any expected value of incorporating technology into a program. In addition to these practical implications, future research should focus on evaluating the impact of strategic planning on the use of technology and how strategic planning may help to facilitate improvements and change.

### 4.2. Limitations

The findings of this study should be interpreted in light of some limitations. Strategic plans were limited to those from Canadian or American accredited pharmacy programs and required the strategic plan to be publicly available on the program’s website. Although it is possible these inclusion criteria may have missed a number of strategic plans, the consistency of our results indicates that similar strategies would be observed across other plans. A second limitation was the difficulty in interpreting the wording or meaning of some strategic plans (e.g., improve vs. innovate vs. introduce). However, the frequent meetings and replication of all coding by independent investigators should have minimized any major misinterpretations and miscoding. Third, this study was limited to education technology and technology for other aspects (e.g., research) was not captured. However, it was noted during the data extraction that few statements were excluded that that did not fit within education. Finally, data were limited to current strategic priorities of programs, and it is possible that strategic planning will naturally shift focus to address the gaps identified by this paper upon evaluation of processes during the COVID-19 pandemic.

## 5. Conclusions

Coming out of the COVID-19 pandemic, pharmacy programs will be entering a “new normal”, which will consist of an elevated baseline of technology use within education systems. As such, programs will need to readdress strategic priorities with respect to using technology as a tool to achieve program goals. The findings of this study support the notion that programs, to date, are focusing on technology as an entity and are not considering human factors that directly influence its implementation and effectiveness. Moving forward, strategic priorities with respect to technology should be refocused towards system readiness and account for resources necessary for target user upskilling and acceptance.

## Figures and Tables

**Table 1:** Strategic priorities for technology in pharmacy education.

Priority	Total (%)	Quote
Acquire	13 (16.5%)	Ensure the required equipment and technology is available to support the delivery of the graduate and undergraduate programs
Introduce/use	22 (27.8%)	Explore and integrate new technologies to enhance course delivery and ensure we have an effective infrastructure for sharing innovations
Improve/expand	21 (26.6%)	Develop a plan to enhance student learning and assessment through advancing technologies to provide students with the tools to be successful both academically and professionally
Prepare	12 (15.2%)	Enhance intramural training for professional staff and faculty in fundamental collaborative technology skills
Evaluate	11 (13.9%)	Examine and clarify the role of technology in our educational programs and develop recommendations for integrating instructional technologies into our curriculum to enhance student learning

**Table 2:** Strategic goals for technology in pharmacy education.

Goal	Sub-Goal	Total (% of All)
Education		56 (70.9%)
	General	12 (15.2%)
	Teach/deliver/learn	27 (34.2%)
	Online/distance courses or degrees	9 (11.4%)
	Other	8 (10.1%)
Practice		8 (10.1)
Communication		7 (8.9%)
Other		5 (6.3%)
Faculty/staff development		3 (3.8%)

## Appendix A

Extracted data from strategic plans:

- Explore and integrate new technologies to enhance course delivery and ensure we have an effective infrastructure for sharing innovations
- We will effectively develop and integrate novel education strategies and technologies to optimize the learning experience.
- The integration of new technologies into our teaching and mentoring of students (measuring/tracking progress)
- Establish institutional priorities to identify credit and non-credit online programs or partially online programs that include flexibility for students to accelerate, decelerate, or remediate. Identify a partner to assist with building online credit and non-credit courses and programs
- Optimize learning through the best use of technology and best-practice teaching strategies
- Ensure that the school has the physical and technical capacity to support leading-edge research and educational programs
- Building from our efforts to support an expanded scope of pharmacist practice, we will leverage our relationships and experience in practice innovation to accelerate change in areas that are fundamental to both optimal patient care and health system sustainability...and technology-enabled care
- Nurture practice innovation in technology-enabled care where relevant for improved patient outcomes
- Ensure the required equipment and technology is available to support the delivery of the graduate and undergraduate programs.
- Identify and support the adoption of tools and strategies that promote best practices in teaching and assessment—establish a technology committee, develop its terms of reference, and support its role within the faculty
- Identify opportunities to use technology to support and enhance teaching
- Increasing cutting edge technology in the learning experience
- Enhancing the information technology infrastructure to facilitate teaching
- Leverage technology and social media for alumni engagement
- Develop and implement a plan to increase technology-assisted learning
- Use technology to enhance teaching and learning
- Implement improved technologies for online testing, advising, and progression
- Faculty trained on online instruction
- Increase online programming for course and degree options. Addition of online programming for course and degree options, including online degree programs and courses.
- Recommend technology enhancements to enable educational best practices
- Promote, enhance, and expand innovative teaching approaches within the SOP, including the use of contemporary teaching technology, simulations, and interprofessional education
- Address adequacy of teaching and research equipment and technology
- Provide or promote awareness of faculty and staff development opportunities in the areas of advising, assessment, development, developmental leave, diversity and inclusivity, leadership, mentoring, outreach, planning, professionalism, promotion and tenure, self-awareness, scholarship and research, technology, and teaching and learning
- Innovation in teaching with a commitment to interprofessional education and practice, simulation, active learning and technology
- Technology in classrooms: Increase technology usage to ensure efficient educational delivery
- Embedding of technology in students’ assignments and presentations
- Expand usage of online resources during class to advance active learning
- Delivery of online examination for applicable courses
- Develop innovative programs that embrace technology to promote interdisciplinary collaboration in providing pharmacy services
- Evaluate and utilize innovative technologies to enhance the educational program
- Engage campus and online students with effective action, and innovative teaching and learning strategies
- Secure a facility with state of the art equipment and technology that will fully support contemporary and forward-thinking education and research
- Develop a plan to create a funding mechanism that would generate $500,000 annually for technology enhancements (IT and research instrumentation) to support research and education
- Establish a task force to identify opportunities for communication and participation through technology and develop a plan to enhance them
- Enhance technology training and support with an objective of creating seamless, high definition, reliable communication
- Enhance communications with all stakeholders via email and other new technologies such as social networks
- Acquire and maintain up-to-date equipment and software and for instructional technology and student assessment
- Develop, enhance and retain appropriate fiscal, human, technological, research and physical resources to achieve the college’s mission
- Encourage the development of online and hybrid courses at all program levels
- Development and implementation of comprehensive distance learning programs. Online training
- Assess and ensure reliable and responsive technical support for teaching, research, and administrative processes
- Implement and disseminate innovative use of technology in patient care—by 2023, assist stakeholders with the evaluation and integration of healthcare technology such as telemedicine into their practice sites
- By 2023, integrate technology into the IPE core in order to provide opportunities for students in various regions to take advantage of distant IPE experiences (such as transplant, oncology, etc.)
- Develop a plan to enhance student learning and assessment through advancing technologies to provide students with the tools to be successful both academically and professionally
- Determine student expectations for utilization of advancing technologies and how they play a role in the ability to learn materials
- Evaluate the role of technology in the pharmacy curriculum (technology with a purpose, not just for show
- To produce pharmacist leaders who effectively apply patient-centered pharmacotherapy, advocacy, and innovative technology
- Develop a system to facilitate support staff members keeping up with new knowledge and technology
- Improve our use of education technologies … by applying and evaluating new education tools, technologies, and methods
- Increase acceptance of technology in teaching and practice
- Develop online and blended courses for enrolled pharmacy students and specialized online CE and certificate programs for current practitioners
- Partner with CETL faculty mentors to help refine pedagogical and technological teaching skills
- Enhance intramural training for professional staff and faculty in fundamental collaborative technology skills
- Explore technology platforms to enhance research, education and outreach infrastructure and delivery
- Evolve our adaptive and responsive curricula to meet practice and research challenges. Continue to innovate and embed learning strategies that will propel learners faster to defined levels of mastery. This includes peer-learning, high-fidelity simulations, portfolios, and technology tools.
- Use a range of technologies and social media to optimize communication with future and current students, alumni and other partners
- Develop online and on-site educational programs for international and domestic professionals
- Develop the needed fiscal, physical, technological and other resources through partnerships with stakeholders and communities to advance our mission
- Large classrooms that can accommodate a full class promote student collaboration and provide adequate support for technology
- Integrate new technology into the delivery of the curriculum and administrative School functions
- Enhance global pharmacy education through distance learning technology and support visiting international scholars
- Adopt the best technologies and practices to enhance a quality, forward-thinking and cost-efficient educational and research environment
- Ensure the adequacy and quality of space and academic technology resources to facilitate learner-centered teaching
- Recommend alterations in teaching facilities and technology, as appropriate to meet the educational needs
- Expand the use of active learning and online learning to enhance student engagement and learning
- Expand graduate programs, including internship opportunities, a global online curriculum and new postgraduate training programs
- Expand distance-based platforms for continuing education
- Secure new space and renovate existing space to meet the needs of the school’s education programs, including modular/flexible space for active learning, simulation and practice-skills facilities, technology-enhanced lecture halls and facilities; contemporary research labs and core support facilities, and informal gathering areas
- Promote lifelong learning through the establishment of professional development and technology training within the college
- Increase the number of online learning experiences throughout the EACPHS
- Examine and clarify the role of technology in our educational programs and develop recommendations for integrating instructional technologies into our curriculum to enhance student learning
